# Regulation of Lipid Metabolism and Beyond

**DOI:** 10.1155/2016/5415767

**Published:** 2016-05-12

**Authors:** Youngah Jo, Hiroaki Okazaki, Young-Ah Moon, TongJin Zhao

**Affiliations:** ^1^Department of Molecular Genetics, University of Texas Southwestern Medical Center, Dallas, TX, USA; ^2^Department of Diabetes and Metabolic Diseases, University of Tokyo, Tokyo, Japan; ^3^Department of Molecular Medicine, Inha University School of Medicine, Incheon, Republic of Korea; ^4^School of Life Sciences, Xiamen University, Xiamen, Fujian, China

One of the worldwide health issues is the increasing number of obese population. In 1975 it was less than 100 million but it reached 600 million in 2014 according to WHO report in 2015. More than one-third of adults and 17% of youth are obese in America [1, 2]. Obesity is one of the major risk factors for diverse human diseases and more and more research papers are coming to dissect the mechanism and find new therapeutic targets. This special issue has been opened to collect recent studies on lipid metabolism including its regulation-dysregulation and new possible biomarkers that can be used for diagnosis of obesity and other related diseases. Also it includes reviews to focus on current studies about regulation mechanism of lipid metabolism.

Lipids play important roles in the body to store energy and as the components of biological membranes, steroid hormones, bile acids, vitamins, and so forth. They are supplied from diets or from the de novo synthesis in the liver. Fatty acids mainly stored as triglycerides are the major energy source for muscle and heart. However, the overproduction and accumulation of triglycerides in adipose tissue and other tissues are closely related to the human metabolic disorders. Disturbance of cholesterol homeostasis is also closely related to atherosclerosis.

The lipids taken from diets are wrapped in chylomicrons to be mobilized to liver or peripheral tissues. Many lines of studies have demonstrated the postprandial hypertriglyceridemia as one of the risk factors for cardiovascular diseases in obese individuals [3]. However it has been unclear why obese patients with polycystic ovarian syndrome (PCOS) have higher incidence of cardiovascular diseases. T. K. Tun et al. have an attention to, in particular, the settings of the postprandial changes in the lipid profile by comparison of two obese groups of PCOS or non-PCOS. The study demonstrates that obese PCOS individuals have higher association with insulin resistance and obesity when compared to the obese control non-PCOS group. Therefore the obesity in polycystic ovary syndrome should be considered as a risk factor and managed to reduce their cardiovascular disease burden.

Another research article demonstrates the one possible biomarker in newly diagnosed type 2 diabetic patients. The role of betatrophin is controversial [4, 5]. M. Yi et al. measured the levels of betatrophin and observed that higher betatrophin level is related to the type 2 diabetes and reversely correlated with the HDL levels. It suggests betatrophin as a new marker for diagnosis of type 2 diabetes in early stage.

Cardiovascular disease (CVD) is a significant health and financial burden to our society that warrants new and more effective therapies. Recent research has discovered novel functions of HDL in the trafficking of microRNAs (miRNAs) as a part of intercellular gene-regulation networks. It has become clear in recent years that miRNAs are one of the many classes of noncoding regulatory small RNAs [6]. Here in this special issue the role of miR-378a has been described. It explains the biological basics and target genes of miR-378a and the roles of miR-378a in lipid metabolism, muscle biology, and proangiogenic effect on blood vessel formation.

Another review article is describing the role of FTO gene, which is associated with obesity in humans and has been identified as the first gene related to obesity in human from the genome-wide association study. The review is explaining how the SNP of FTO gene in intron 1 regulates the expression of various genes to increase adiposity in various tissues during developmental stages.

As the role of adipose tissue has shed light on the studies of obesity-related diseases, one review article is demonstrating the role of various adipokines in many metabolic diseases. E. Kantorová et al. have summarized the role of adipokines in neurological diseases.

The complication of dyslipidemia is also related to the inflammatory processes involved in the pathogenesis of atherosclerosis. M. Koren-Gluzer et al. are demonstrating the anti-inflammatory role of paraoxonase 2 in induction of polarization of macrophage to M2 state using a knockout mice model of paraoxonase 2.

There are many factors that regulate lipid metabolism. This special issue is representing basic research and clinical works using a small number of samples and reviews of current studies. However it will provide a line of data to make many people understand the mechanism of lipid regulation and to develop new possible biomarkers or drugs to advance our human life.

*Youngah Jo* *Youngah Jo* *Hiroaki Okazaki* *Hiroaki Okazaki* *Young-Ah Moon* *Young-Ah Moon* *TongJin Zhao* *TongJin Zhao*
